# Inhibition, transition, and surge: dynamic evolution of pediatric respiratory pathogen trends amid COVID-19 pandemic policy adjustments

**DOI:** 10.3389/fpubh.2024.1420929

**Published:** 2024-08-22

**Authors:** Xuena Xu, Zhenzhen Pan, Heting Dong, Yizhu Zhang, Lina Xu, Pengli Wang, Yufeng Wang, Jiaoyang Li, Wujun Jiang, Chuangli Hao

**Affiliations:** ^1^Department of Respiratory Medicine, Children’s Hospital of Soochow University, Suzhou, China; ^2^Department of Respiratory Medicine and Clinical Allergy Center, Affiliated Children’s Hospital of Jiangnan University, Wuxi, China

**Keywords:** acute respiratory tract infections, COVID-19 pandemic, respiratory pathogens, children, pandemic policy adjustments

## Abstract

**Background:**

The implementation of a zero-COVID policy for 3 years in China during the COVID-19 pandemic significantly impacted a broad spectrum of acute respiratory tract infections (ARTIs). The epidemiological characteristics of ARTI pathogens in children following the cessation of the zero-COVID policy remain unclear.

**Methods:**

Etiologically diagnostic data from 82,708 children with ARTIs at the Children’s Hospital of Soochow University during 2016–2023 were analyzed for 8 pathogens (human respiratory syncytial virus [HRSV], influenza A [FluA], FluB, human parainfluenza virus [HPIV], adenovirus [ADV], human rhinovirus [HRV], bocavirus [BoV], and *mycoplasma pneumoniae* [MP]). The changes in respiratory infections in Suzhou, China during the first year (2020, Phase I) and the second and third years of the pandemic (2021–2022, Phase II) and the first year after the end of zero-COVID policy (2023, Phase III) versus that in the pre-pandemic years (2016–2019) were compared.

**Results:**

When compared with the average pre-pandemic levels, the pathogen-positive rate decreased by 19.27% in Phase I (OR: 0.70; 95% CI: 0.67–0.74), increased by 32.87% in Phase II (OR: 1.78; 95% CI: 1.72–1.84), and increased by 79.16% in Phase III (OR: 4.58; 95% CI: 4.37–4.79). In Phase I, the positive rates of HRSV, FluA, ADV, and MP decreased by 26.72, 58.97, 72.85, and 67.87%, respectively, and the positive rates of FluB, HPIV, HRV, and BoV increased by 86.84, 25, 32.37, and 16.94%, respectively. In Phase III, the positive rates of HRSV, FluA, FluB, HPIV, ADV, and HRV increased by 39.74, 1046.15, 118.42, 116.57, 131.13, and 146.40%, respectively, while the positive rate of BoV decreased by 56.12%. MP was inhibited during the epidemic, and MP showed a delayed outbreak after the ending of the zero-COVID policy. Compared with the average pre-pandemic levels, the MP-positive rate in Phase III increased by 116.7% (OR: 2.86; 95% CI: 2.74–2.99), with the highest increase in 0–1-year-old children.

**Conclusion:**

The strict and large-scale implementation of the zero-COVID policy in the early stages of the COVID-19 pandemic was the main driving factor for the sharp reduction in the rate of children’s respiratory pathogenic infections. The termination of this policy can cause a resurgence or escalation of pathogenic infections.

## 1 Introduction

In December 2019, the world witnessed the emergence of pneumonia cases caused by SARS-CoV-2 in Wuhan, China (1). This marked the beginning of a global health crisis, with the World Health Organization declaring COVID-19 a pandemic on March 11, 2020 (2). In response to the escalating situation, China implemented a zero-COVID policy on January 24, 2020, characterized by stringent measures including lockdowns, restrictions on gatherings, home isolation, suspension of education and work, as well as hygiene practices such as handwashing and mandatory mask usage (3). These non-pharmaceutical interventions (NPIs) played a pivotal role in not only curbing the spread of COVID-19 but also in altering the epidemiology of various respiratory pathogens (4–8). The transmission dynamics of respiratory pathogens are complex and influenced by a multitude of factors including environmental conditions, climatic variations, and population movements (9, 10). As regions begin to relax COVID-19 restrictions, there is a noticeable resurgence of certain respiratory pathogens (11–13).

China’s decision to end its zero-COVID policy on December 27, 2022, was based on the decreased pathogenicity of the Omicron variant and the higher national vaccination rates. However, this marked the onset of a rapid nationwide COVID-19 outbreak, signifying the conclusion of its extensive pandemic containment efforts (14). Subsequently, diseases such as human respiratory syncytial virus (HRSV) and influenza exhibited atypical seasonal outbreaks (15, 16). Prolonged social distancing measures reduced exposure to respiratory pathogens, leading to a decline in population immunity and a surge in cases, commonly referred to as “immunity debt” (17, 18). Modeling studies conducted in South Africa, the Netherlands, and Japan have raised concerns about the potential HRSV epidemics upon the abolition of restrictive measures, attributed to weakened immunity during the pandemic (19–21). Baker et al. predicted through digital models that prolonged implementation of NPIs may coincide with seasonal peaks, resulting in larger future outbreaks of HRSV and influenza on average (22). A digital model study in the United Kingdom projected that post the cancelation of NPIs if exposure levels return to pre-pandemic norms, the expected annual incidence of norovirus might double compared with that in typical years (23). Unlike other countries, China’s 3-year implementation of restrictive measures may have led to excessive population protection and a more pronounced immune debt burden.

Acute respiratory tract infections (ARTIs) pose a significant health burden on children, as evidenced by the elevated rates of associated hospitalization and mortality (24). Respiratory pathogens, including respiratory viruses and *mycoplasma pneumoniae* (MP), play a crucial role in the development of ARTIs. A nationwide prospective study in China showed a viral positive rate of 46.9% in children under 5 years of age and in school-age children. The three leading viral pathogens identified were influenza virus (28.5%), HRSV (16.8%), and human rhinovirus (HRV) (16.7%) (25). In addition, MP is a prevalent cause of respiratory infections and is recognized as the primary pathogen responsible for community-acquired pneumonia in children (26, 27). Nevertheless, the common pathogenic trend among children with ARTIs in China after the end of the zero-COVID policy remains to be fully explored.

This study aims to address this gap by conducting a retrospective cohort analysis using data from pediatric ARTI cases. We seek to elucidate the impact of COVID-19 policy adjustments on pediatric respiratory pathogen epidemiology. The insights gleaned from this study are crucial for formulating effective public health strategies and managing the resurgence of respiratory pathogens.

## 2 Materials and methods

### 2.1 Study population

**Figure 1:**
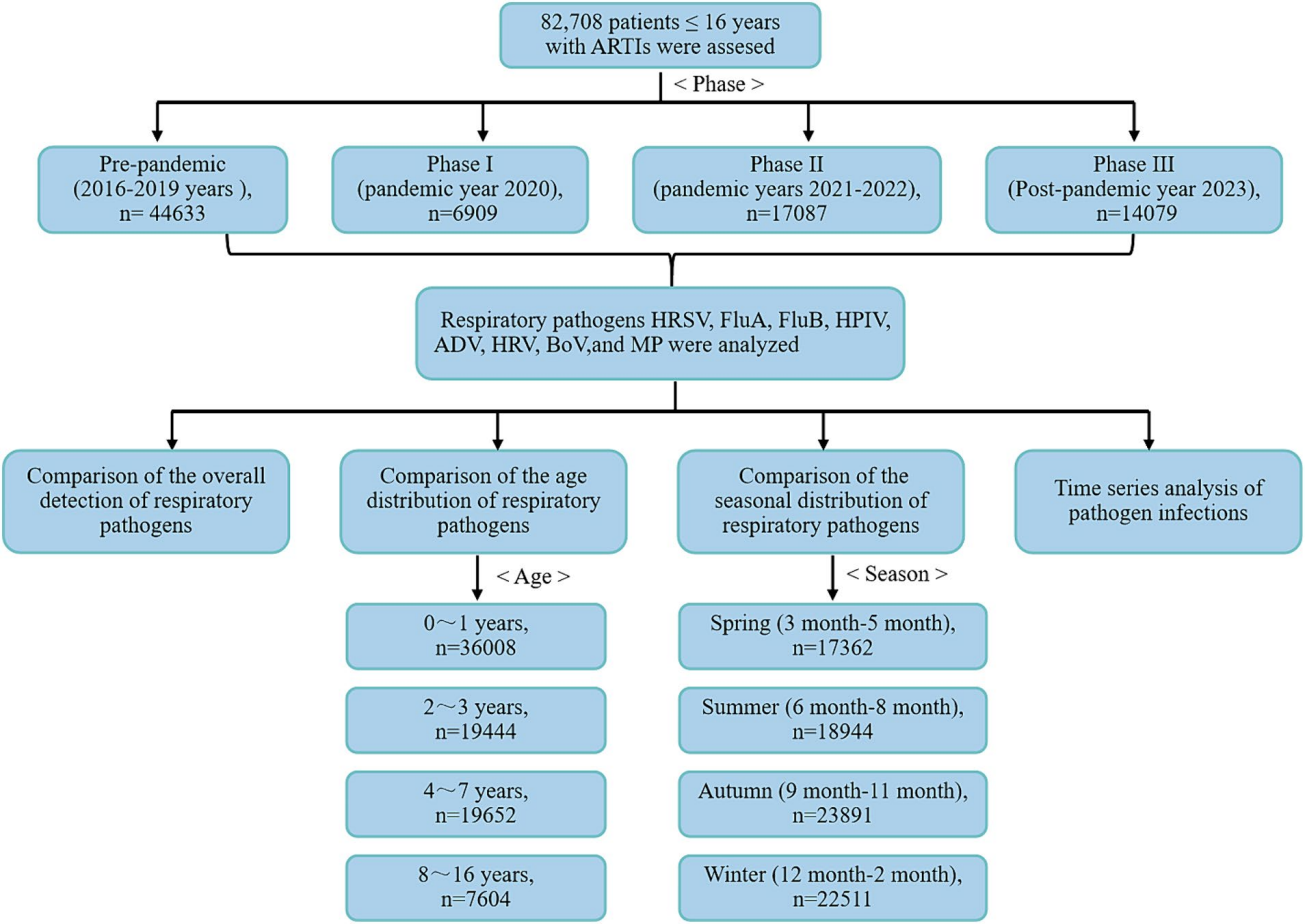
Flowchart depicting the process of selection of the study participants in the current analysis.

We analyzed data from 82,078 pediatric patients with ARTIs aged 16 years or younger who were hospitalized at the Children’s Hospital of Soochow University between January 2016 and December 2023 (Figure 1). The Children’s Hospital of Soochow University, Suzhou, Southeast China, serves as the primary tertiary children’s hospital for the region. ARTIs in children were diagnosed by experienced physicians using specific criteria, including fever (ear temperature ≥ 37.5°C), presence of at least one respiratory symptom in the previous 14 days (e.g., cough, sore throat, rhinorrhea, sneezing, rapid or difficult breathing), and the need for hospitalization. To ensure data accuracy, rigorous exclusion criteria were applied, including the exclusion of patients with multiple clinic visits within the past 1 week, those with hospital-acquired infections, and individuals with COVID-19 infection. These patients were tested for the presence of the following 8 pathogens, including HRSV, influenza A (FluA), influenza B (FluB), human parainfluenza virus (HPIV), adenovirus (ADV), HRV, bocavirus (BoV), and MP.

To illustrate the impact of pandemic policy adjustments on pathogen circulation patterns, we defined four periods from 2016 to 2023 based on the implementation and relaxation of the zero-COVID policy in Suzhou: January 2016 to December 2019 (pre-pandemic), January 2020 to December 2020 (Phase I), the first year of COVID-19 in Suzhou, January 2021 to December 2022 (Phase II)—during this period national NPIs were gradually relaxed while the dynamic zero-clearing policy was introduced, and, finally, January–December 2023 (Phase III), representing the first year after China terminated the zero-COVID policy (Supplementary Figure 1). Additionally, the study compared and analyzed the positive rates of respiratory pathogens across different age groups and seasons. The study received approval from the Medical Ethics Committee of the Children’s Hospital of Soochow University (2013002).

### 2.2 Respiratory pathogen detection

Nasopharyngeal aspirates were carefully collected from patients within 24 h of admission using a straw gently inserted through the nasal passage to reach the lower part of the throat at a depth of 7–8 cm. Following collection, approximately 1–2 mL of nasopharyngeal aspirate was immediately mixed with a 0.9% sodium chloride solution and swiftly transported to the laboratory for analysis within 30 min. Stringent quality control measures were employed, whereby samples with fewer than 10 squamous epithelial cells and more than 20 white blood cells per low magnification field under an optical microscope were deemed unqualified and either resampled or discarded. Subsequently, the samples underwent centrifugation at 500 × *g* for 10 min, and the resulting supernatant was resuspended in 2 mL of physiological saline for the detection of various respiratory pathogens. Total nucleic acid (comprising DNA and RNA) was extracted from these samples, followed by analysis using a commercial multiplex polymerase chain reaction-based panel assay (Health Gene Technologies, Ningbo, China) to identify common respiratory pathogens, including HRSV, FluA, FluB, HPIV, ADV, HRV, BoV, and MP. Detailed information regarding the primer/probes used and amplification conditions was available upon request. Throughout the nucleic acid extraction and PCR/RT-qPCR testing process, positive and negative controls were used to ensure accuracy and reliability.

### 2.3 Statistical analysis

Categorical variables were comprehensively reported as frequencies and percentages. Group comparisons were conducted using either the chi-square test or Fisher’s exact probability method. Univariate binary logistic regression was performed to calculate the odds ratios (ORs) and 95% confidence intervals (CIs). ORs for the odds of positive respiratory pathogen test was conducted between Phases I, II, and III and the pre-pandemic period. The percentages of positive pathogen tests during Phases I, II, and III were compared with the pre-pandemic average levels by calculating the percentage change using the following formula, stratified by age groups:

$\left(P_{pha}-P_{pre}\right)/P_{pre}\times 100\%$

where *P*_pha_ represents the average positive rate during Phase I, Phase II, or Phase III, while *P*_pre_ denotes the average positive rate in the pre-pandemic period (2016–2019). Additionally, a time series analysis was conducted based on the monthly positive rates from 2016 to 2019 to predict the corresponding rates for 2020 to 2023, along with their corresponding 95% CIs. Linear models were employed based on the observed patterns of changes in the positive rates prior to 2020 and the associated model fit indices.

Database establishment and analyses were performed using SPSS 27.0 (SPSS Inc., Chicago, IL, USA), GraphPad Prism v9 (GraphPad Software, San Diego, CA), and Origin Pro 2019 (OriginLab Corporation, Northampton, MA, USA). Statistical significance was set at *p* < 0.05.

## 3 Results

### 3.1 General clinical characteristics

The study encompassed a total of 82,708 patients with ARTIs between January 1, 2016, and December 31, 2023. Among these patients, 44,633 cases (53.96%) were diagnosed during the pre-pandemic period, 6,909 cases (8.35%) during Phase I, 17,087 cases (20.66%) in Phase II, and 14,079 cases (17.02%) in Phase III (Table 1). Statistical analysis revealed significant sex differences in both Phase II and Phase III compared with the pre-pandemic period (*p* < 0.05). Additionally, the hospitalization rate in the ICU during Phase II significantly increased compared with pre-pandemic years (4.49% vs. 3.85%, *p* < 0.05). The overall positive rate of respiratory viruses was 38.37%, with HRSV (10.40%) being the most prevalent respiratory virus. The positive rate of MP was 13.22% (Supplementary Figure 2). Between 2016 and 2023, the yearly counts of tested cases were 7,193, 8,435, 11,846, 17,159, 6,909, 9,273, 7,814, and 14,079. The corresponding annual numbers of positive cases were 3,044, 3,561, 4,926, 7,875, 2,425, 4,692, 5,180 and 10,968 (Supplementary Table 1).

**Table 1:** Comparison of the demographics and positive rates (%) of respiratory pathogens across pre-pandemic, Phase I, Phase II, and Phase III periods.

	Total	Pre-pandemic	Phase I	Phase II	Phase III
Demographics					
Total patients	82,708	44,633	6,909	17,087	14,079
Male sex, *n* (%)	47,344 (57.24)	25,977 (58.20)	4,065 (58.83)	9,735^#^ (56.97)	7,567^★^ (53.75)
Age, *n* (%)					
0 ~ 1 years	36,008 (43.54)	22,887 (51.28)	3,385^*^ (48.99)	6,631^#^ (38.81)	3,105^★^ (22.05)
2 ~ 3 years	19,444 (23.51)	10,219 (22.90)	1,779^*^ (25.75)	4,865^#^ (28.47)	2,581^★^ (18.33)
4 ~ 7 years	19,652 (23.76)	9,016 (20.20)	1,297^*^ (18.77)	4,231^#^ (24.76)	5,108^★^ (36.28)
8 ~ 16 years	7,604 (9.19)	2,511 (5.63)	448^*^ (6.48)	1,360^#^ (7.96)	3,285^★^ (23.33)
Pathogen detection, *n* (%)					
HRSV	10,363 (12.53)	5,112 (11.45)	580^*^ (8.39)	2,417^#^ (14.15)	2,254^★^ (16.01)
FluA	1,992 (2.41)	349 (0.78)	22^*^ (0.32)	362^#^ (2.12)	1,259^★^ (8.94)
FluB	638 (0.77)	171 (0.38)	49^*^ (0.71)	301^#^ (1.76)	117^★^ (0.83)
HPIV	3,985 (4.82)	1,484 (3.32)	287^*^ (4.15)	1,202^#^ (7.03)	1,012^★^ (7.19)
ADV	1,426 (1.72)	673 (1.51)	28^*^ (0.41)	233 (1.36)	492^★^ (3.49)
HRV	10,612 (12.83)	3,847 (8.62)	788^*^ (11.41)	2,986^#^ (17.48)	2,991^★^ (21.24)
BoV	4,079 (4.93)	2,185 (4.90)	396^*^ (5.73)	1,196^#^ (7.00)	302^★^ (2.15)
MP	14,754 (17.84)	7,686 (17.22)	382^*^ (5.53)	1,432^#^ (8.38)	5,254^★^ (37.32)
Positive case	42,671 (51.59)	19,406 (43.48)	2,425^*^ (35.10)	9,872^#^ (57.77)	10,968^★^ (77.90)
Pneumonia cases	68,619 (82.97)	38,887 (87.13)	5,451^*^ (78.90)	12,868^#^ (75.31)	11,413^★^ (81.06)
ICU admissions	3,351 (4.01)	1,718 (3.85)	294 (4.26)	768^#^ (4.49)	571 (4.06)

^*^p < 0.05 indicates significant differences between the positive rates during Phase I and the pre-pandemic positive rates. ^#^*p* < 0.05 indicates significant differences between the positive rates during Phase II and the pre-pandemic positive rates. ^★^*p* < 0.05 indicates significant differences between the positive rates during Phase III and the pre-pandemic positive rates. Positive cases include both single and mixed respiratory infections.

### 3.2 Overall detection of respiratory pathogens

**Figure 2:**
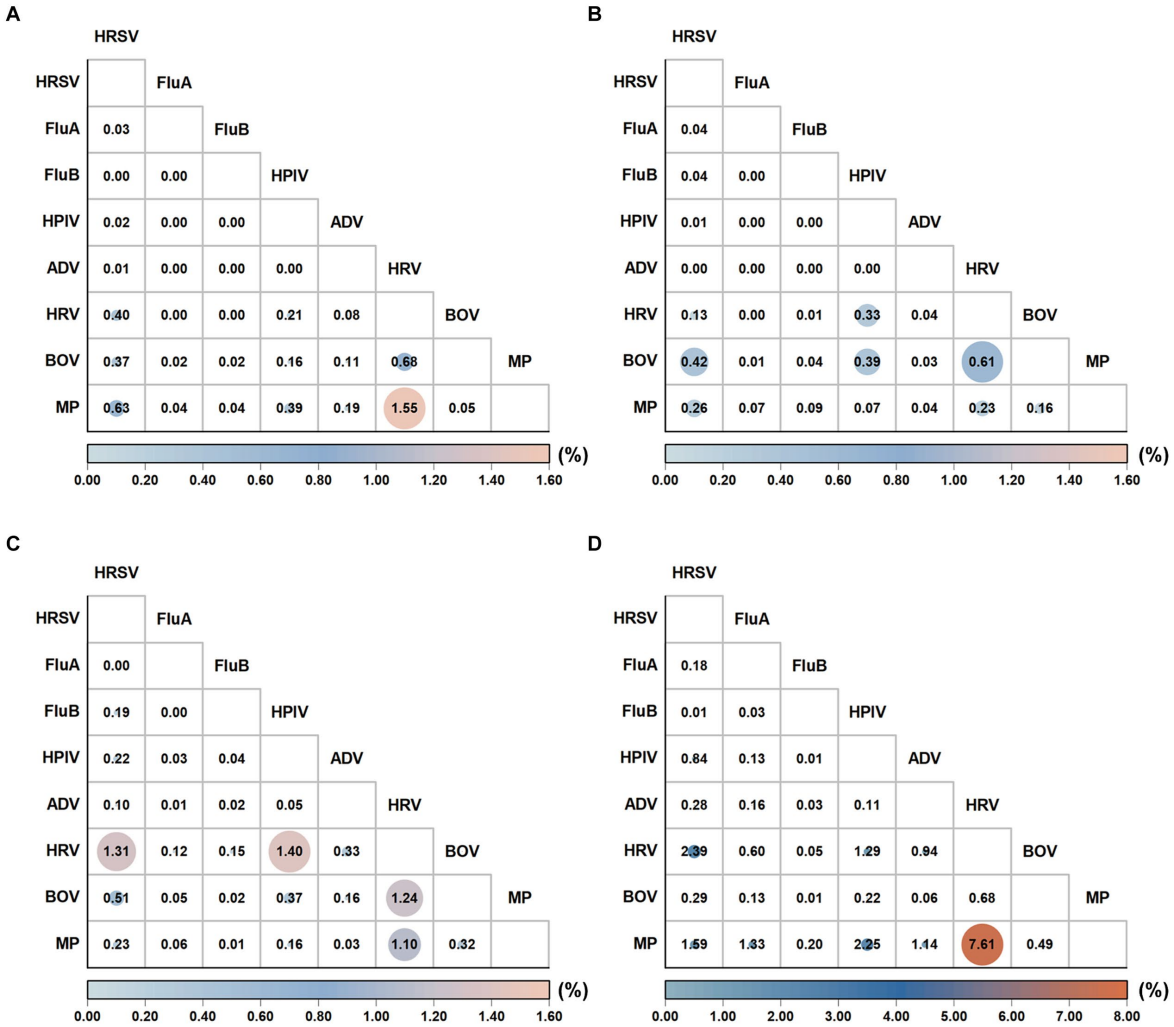
Comparison of the coinfection patterns across pre-pandemic, Phase I, Phase II, and Phase III periods. The coinfection rates (%) were calculated pairwise for pathogens ‘X’ and ‘Y.’ The numerator represents the number of patients coinfected with both ‘X’ and ‘Y’, while the denominator encompasses the total number of patients who were tested for both ‘X’ and ‘Y.’ The resulting figure illustrates the coinfection rate (%) for pathogens ‘X’ and ‘Y.’ The coinfection pattern during pre-pandemic **(A)**, Phase I **(B)**, Phase II **(C)**, and Phase II **(D)**.

The pathogen positive rates in the analyzed samples showed the following changes from the pre-pandemic years to Phase III: The positive rate was 43.48% (19,406/44,633) before the pandemic and decreased to 35.10% (2,425/6,909) in Phase I. Subsequently, it significantly increased to 57.77% (9,872/17,087) in Phase II and peaked at 77.90% (10,968/14,079) in Phase III (Table 1). MP was the most predominant pathogen in both the pre-pandemic period and Phase III, accounting for 39.61% (7,686/19,406) of positive samples in the pre-pandemic period and 47.90% (5,254/10,968) in Phase III. Notably, the MP positive rate during Phase III exhibited a more significant increase than in the pre-pandemic period (37.32% vs. 17.22%, *p* < 0.05). HRV was the most predominant pathogen in Phases I and II, accounting for 32.49% (788/2,425) of positive cases in Phase I and 30.25% (2,986/9,872) in Phase II. An analysis of pathogen positive rates across the four defined periods revealed that HRSV, FluA, HPIV, ADV, HRV, and MP exhibited their peak positive rates in Phase III. Conversely, the lowest positive rates for HRSV, FluA, ADV, and MP were recorded during Phase I. When comparing the positive rates of pathogens (HRSV, FluA, FluB, HPIV, HRV, BoV, and MP) from Phases I, II, and III to pre-pandemic levels sequentially, statistically significant changes were observed in each phase (*p* < 0.05). In Phase III, the number of ARTI cases and positive rates consistently remained high from March to December (Supplementary Figure 1). Supplementary Figure 3 further illustrates the monthly number of positive cases and monthly positive rates for each respiratory pathogen. Most respiratory pathogens were significantly suppressed during Phase I due to the implementation of the zero-COVID policy in China. Specifically, HRSV, FluA, FluB and ADV showed varying degrees of infection gaps. In Phase III, an outbreak of FluA occurred from March to April 2023, while the number of case and the positive rate of HRSV peaked in May 2023. In addition, the positive case and positive rate of MP show a monthly increasing trend. Additionally, during Phase I, coinfections with two or more pathogens decreased, while in Phase III, these coinfections increased. The coinfection rate of MP and HRV in Phase III was as high as 7.61% (Figure 2 and Supplementary Table 2).

### 3.3 Comparison of age distribution

**Figure 3:**
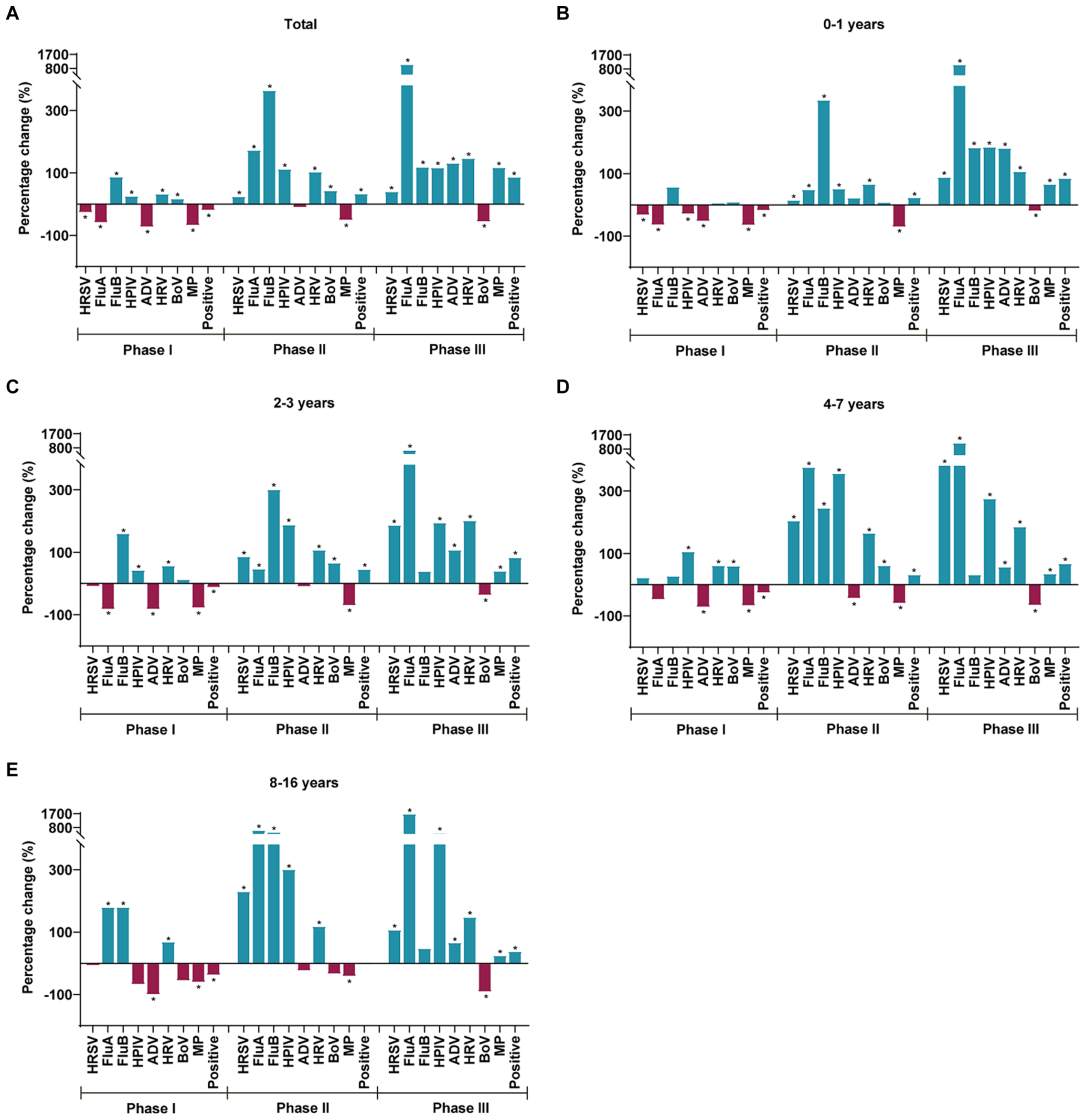
Percentage change in the test positive rate for respiratory pathogens during Phases I, II, and III when compared to the average positive rates in the pre-pandemic, as stratified by the age group **(A–E)**. Blue and red bars indicate positive and negative percentage changes, respectively. Chi-square test was used to compare respiratory pathogen positive rates in Phases I, II and III with those in the pre-pandemic period according to age stratification, with *p < 0.05 defined as statistically significant.

Patients were categorized into four age groups. The highest positive case-positive rate was observed in children aged 8–16 years (54.88%) during the pre-pandemic period, and it shifted to children aged 2–3 years in Phase I (40.13%), Phase II (66.04%), and Phase III (82.99%). Statistically significant differences were noted in all eight respiratory pathogens’ positive rates among the four age groups pre-pandemic, in Phase I, Phase II, and Phase III (*p* < 0.05) (Supplementary Table 3). Figure 3 depicts the percentage change in the test-positive rate of various respiratory pathogens during Phases I, II, and III when compared with the average positive rates during the pre-pandemic years and stratified by age group. In Phase I, the positive rates of HRSV, FluA, ADV, and MP notably decreased by 26.72, 58.97, 72.85, and 67.87%, while the positive rates of FluB, HPIV, HRV, and BoV significantly increased by 86.84, 25, 32.37, and 16.94%, respectively. During Phases II and III, the activity of HRSV, FluA, FluB, HPIV, and HRV surged above historical levels, with the increase in the positive rate of positive cases in Phase III greater than that in Phase II (Figure 3A). In Phases II and III, the positive rates of HRSV, FluA, HPIV, and HRV in all age groups surpassed those in the pre-pandemic period. Specifically, in Phase III, the positive rate of BoV exhibited a significant decrease across all age groups. Notably, MP faced widespread suppression in Phases I and II but significantly resurged in Phase III across all ages, particularly in children aged 0–1 years (Figures 3B–E).

**Figure 4:**
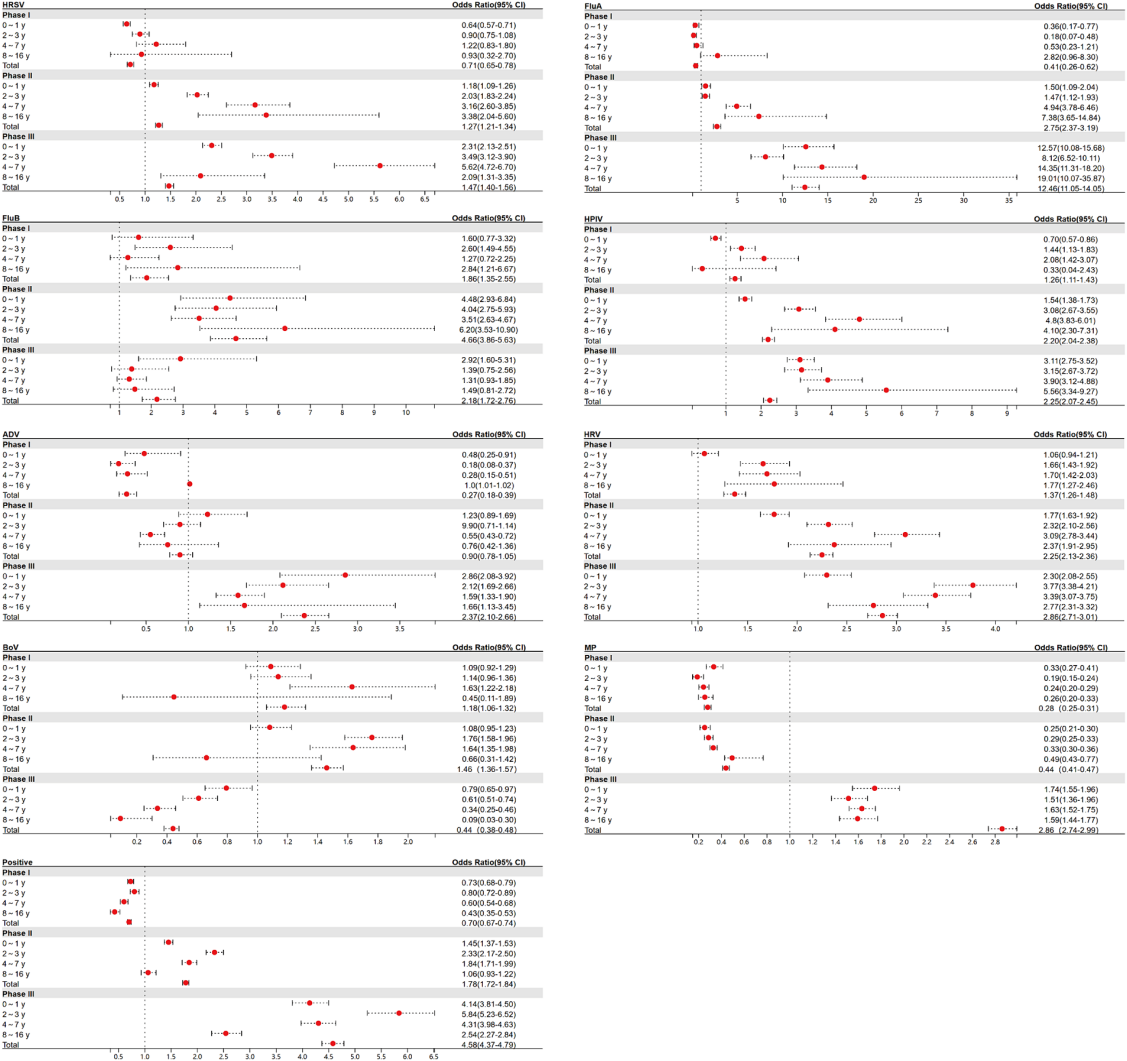
Estimated odds ratios (ORs) for the odds of a positive test between Phases I, II, and III and pre-pandemic for each of the 8 respiratory pathogens and positive cases.

For children in each age group, ORs of respiratory pathogen positive rate between Phases I, II, and III and the pre-pandemic period were calculated (Figure 4). The pattern of these phase-specific ORs largely mirrored the percentage changes shown in Figure 3, with some minor deviations. During Phase I, the ORs of the total age group for pathogens such as MP and positive cases were observed to be less than 1. The significant resurgence peaks of most pathogens occurred in Phase III in the following groups: HRSV in children aged 4–7 years (OR: 5.62; 95% CI: 4.72–6.70), FluA in children aged 8–16 years (OR: 19.01; 95% CI: 10.07–35.87), HPIV in children aged 8–16 years (OR: 5.56; 95% CI: 3.34–9.27), ADV in children aged 0–1 years (OR: 2.86; 95% CI: 2.08–3.92), HRV in children aged 2–3 years (OR: 3.77; 95% CI: 3.38–4.21), MP in children aged 0–1 years (OR: 1.74; 95% CI: 1.55–1.96), and positive cases in children aged 2–3 years (OR: 5.84; 95% CI: 5.23–6.52). FluB, with an OR over 1 in Phase I, saw its peak increase in Phase II within children aged 8–16 years (OR: 6.20; 95% CI: 3.53–10.09).

### 3.4 Seasonal distribution of respiratory pathogens

**Figure 5:**
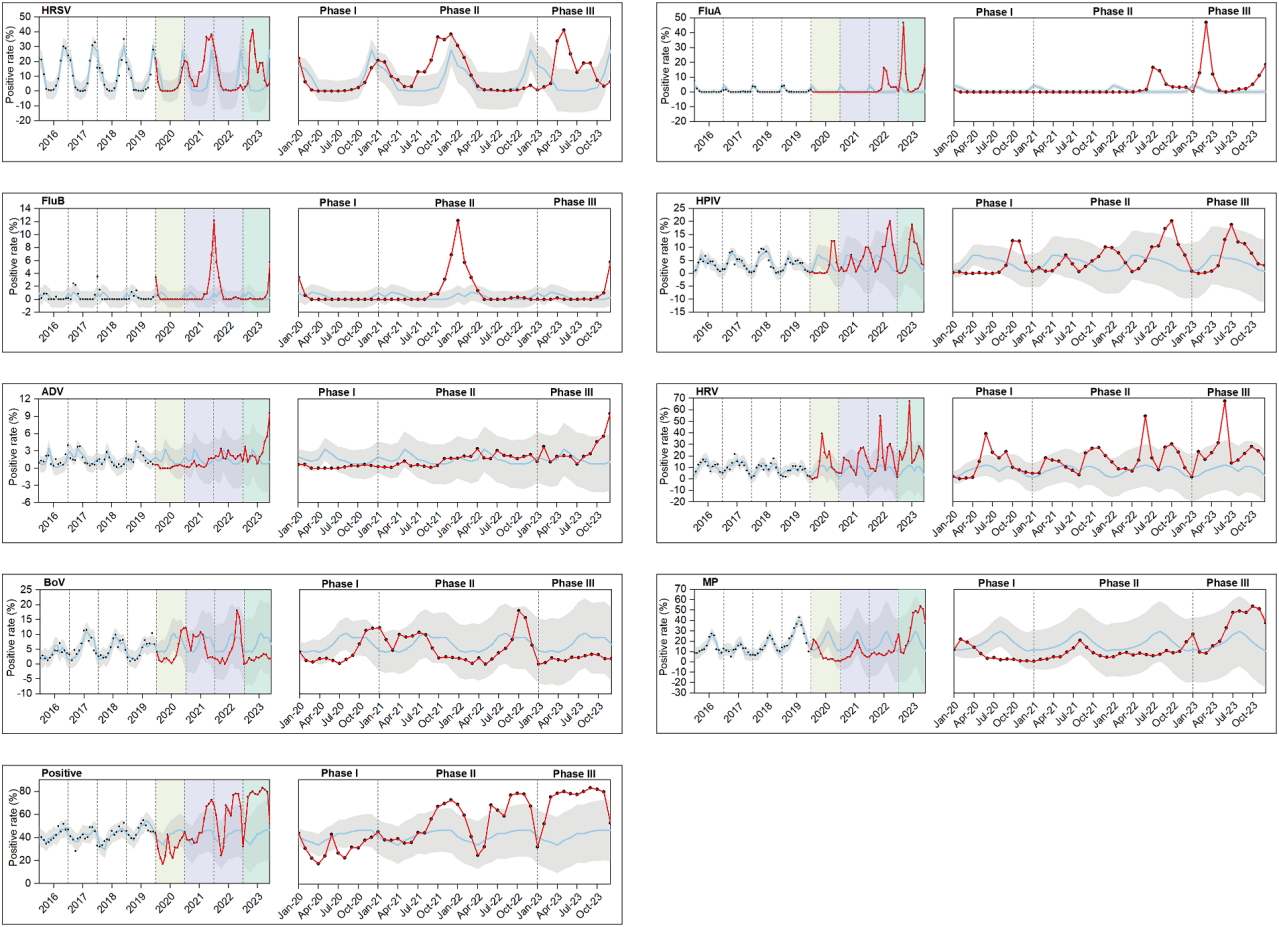
Observed and model-fitted time series of monthly numbers of test-positive rates during 2016–2023. A total of 8 respiratory pathogens were investigated: HRSV, FluA, FluB, HPIV, ADV, HRV, BoV, and MP. Positive include both single and mixed respiratory infections. The hypothetical rates of positive samples (blue line) from January 2020 to December 2023 in the absence of the COVID-19 pandemic were projected using the model based on January 2016 to December 2019. The red line represents pathogen positivity trends during Phases I, II, and III. Phase I (January–December 2020) is indicated in pale yellow, Phase II (January 2021 to December 2022) in lavender, and Phase III (January–December 2023) in green. The confidence bands are depicted in gray, and the observed data points are marked with black dots. For each pathogens, the images during Phases I, II, and III were separately magnified for improved readability.

Utilizing data from 2016 to 2019, we conducted a time series analysis to forecast and fit pathogen positive rates for 2020–2023 (Figure 5). Typically, pathogen positive rates followed stable seasonal trends before the pandemic. However, during Phase I, these trends were disturbed, with peaks for pathogens that usually occurred in late winter, spring, or early summer being suppressed or delayed, leading to no cases of HRSV being reported from April to July 2020 and no cases of FluA being reported from February 2020 to April 2022. By Phase II, except for ADV and MP, most pathogens had rebounded or exceeded the pre-pandemic average level, with FluB experiencing a short-term outbreak. Nevertheless, a complete restoration of their seasonal patterns had not yet been achieved by Phase III. The positive rates of various pathogens were compared across four stages within the same season. Most pathogens showed significant differences in the positive rates between stages (*p* < 0.05), except for FluB, which did not exhibit significant differences in summer positive rates across stages (Supplementary Table 4).

**Figure 6:**
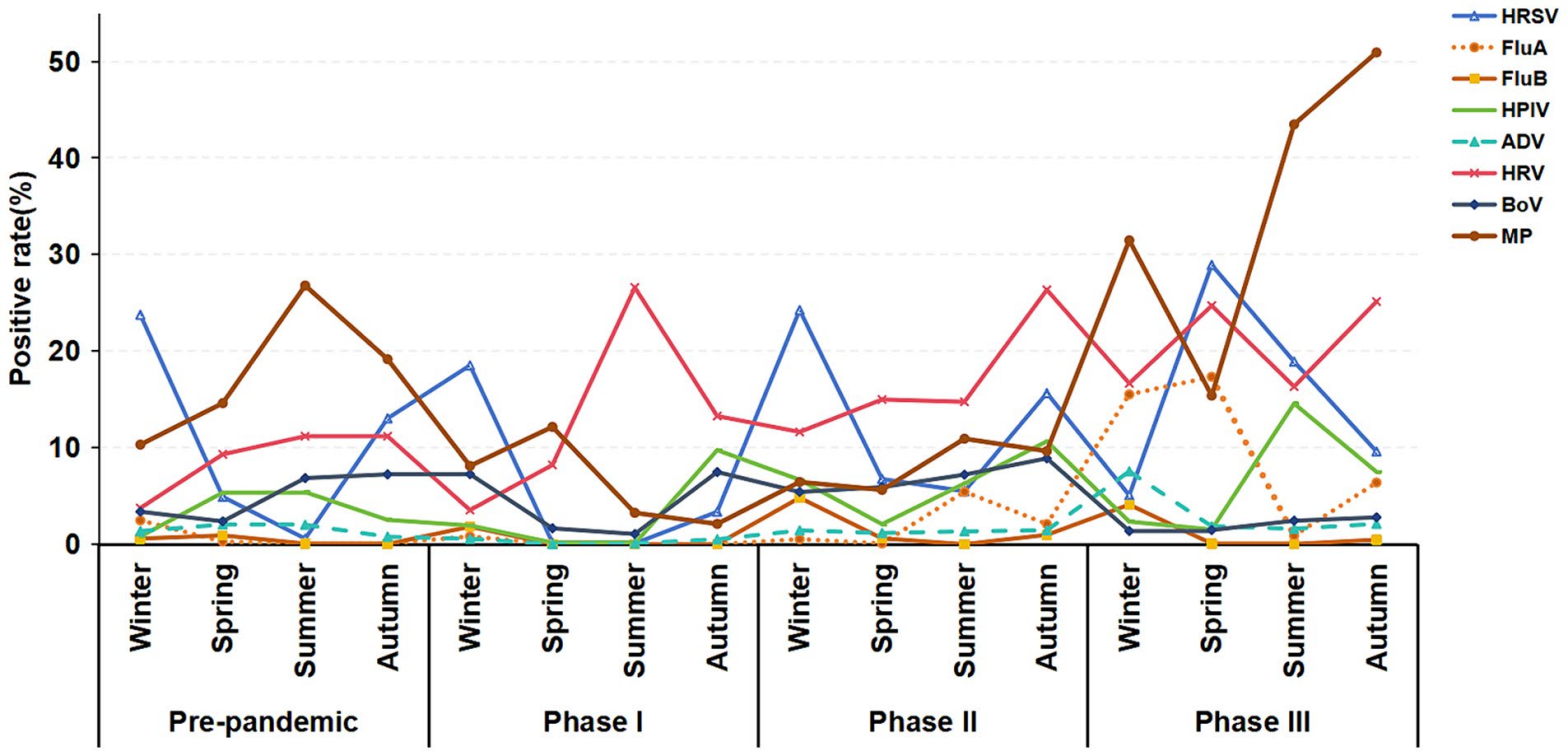
Seasonal distribution trends of respiratory pathogen infections in hospitalized children with ARTIs across the pre-pandemic, Phase I, Phase II, and Phase III periods.

Figure 6 further shows that in Phase I and II and the pre-pandemic period, the winter positive rates for HRSV were higher than other season positive rates. Additionally, the positive rate of HRSV (28.92%) in spring was significantly higher than that in other seasons during Phase III. Furthermore, the positive rates for FluA in winter (15.51%), spring (17.34%), and autumn (6.39%) during Phase III were higher than the positive rates of the same season in other periods. The peak positive rate of ADV occurred in the winter (7.54%) of Phase III. The positive rates for MP in the summer (43.48%) and autumn (50.53%) during Phase III were higher than the rates observed in the same seasons pre-pandemic (summer: 26.27%, autumn: 19.16%).

## 4 Discussion

Understanding the transmission dynamics of respiratory pathogens in children alongside COVID-19 is crucial for assessing the overall impact of the pandemic and related containment measures on children’s health. In this study, we analyzed data from 82,708 Chinese children suspected of respiratory pathogen infections from 2016 to 2023. To our knowledge, this was the first large-scale study in China to evaluate changes in the pattern of respiratory pathogen infections in children across the three phases of the COVID-19 pandemic. We found that while the implementation of the zero-COVID policy changed the transmission trend of some respiratory pathogens, the main types of pathogens in children with ARTIs remained largely unchanged, with MP, HRSV, and HRV continuing to be the main infectious agents.

HRSV and FluA have been major concerns globally before, during, and after the COVID-19 pandemic. Our research indicates that during Phase I, especially in the initial stage when the zero-COVID policy was most strictly implemented, the activities of many pathogens were inhibited, resulting in a prolonged period of infection blank for HRSV and FluA. HRSV is a significant cause of hospitalization and mortality in infants and young children worldwide, particularly in resource-limited countries (28–30). Consistent with previous studies, we observed that HRSV was predominantly prevalent in winter before the COVID-19 epidemic, with a high positive rate among infants (31, 32). Furthermore, HRSV becomes inactive during the strict implementation of NPIs (7, 33, 34). Moreover, several countries have reported seasonal changes and delayed outbreaks of HRSV during the pandemic (35–40). For example, the outbreak of HRSV in Taiwan, China, in 2020–2021 was delayed compared to previous seasons, with an increase in cases among children over 2 years old (41). Similarly, data from the United States showed an early end to the 2019–2020 flu season and no flu outbreaks in the 2020–2021 season (42).

This study revealed that during Phase II, as COVID-19 vaccination gained momentum in China and the public became accustomed to epidemic measures, the government gradually eased mitigation measures such as home quarantine and lockdowns. This policy adjustment led to a swift resurgence of other respiratory pathogens, notably HRSV and FluA, surpassing positive rates even before the pandemic. Li et al. observed, in an analysis across 18 countries, that the full reopening of schools was associated with an increased risk of HRSV rebound, with the risk trending upward over time (43). In our previous studies, we noted a 70.7% increase in the HRSV-positive rate in 2021, particularly notable in children aged 2–4 years (38). In Phase III, particularly following the initial relaxation of the zero-COVID policy, China faced a short-term COVID-19 outbreak. A study in Macau, China, indicated that 70% of the population contracted COVID-19 within the first 3 weeks after the policy relaxation (14). During this period, our time series data indicated a decline in the positive rates of various respiratory pathogens, possibly due to individuals opting for self-treatment at home, leading to reduced mobility and hospital visits. However, as mobility and social activities resumed, respiratory pathogens exhibited a strong resurgence. From February to December 2023, the monthly positive rate of respiratory pathogens consistently exceeded 50%, with an annual positive rate of 77.90%. The increase in the FluA positive rate even reached 1046.15%. Liu et al. identified a FluA outbreak in Shanghai, China, post-easing of the zero-COVID policy in February–March 2023 (15). Russia also reported an epidemic of FluA (H1N1) pdm09 viruses following the COVID-19 pandemic (44). An Italian study highlighted a significant outbreak of “pure” HRSV in the first year after the relaxation of social containment measures, with consistent clinical manifestations observed in the subsequent two seasons (45). In the study, we noted a shift in the epidemics of HRSV, with an increase in the positive rates across all age groups, particularly among infants and young children in 2023. Furthermore, our study results suggest that the positive rate of HRSV was highest in winter before and during the COVID-19 pandemic, whereas in Phase III, the positive rate of HRSV was highest in spring. The highest positive rate of FluA during the epidemic was seen in winter. Phase I did not exhibit a clear seasonal pattern for FluA due to its significant inhibition. The positive rate of FluA in Phase II was highest in summer, while in Phase III, it was highest in spring. These findings indicate that the measures implemented in our COVID-19 policy have influenced the seasonal positive rate of HRSV and FluA. Even as normal daily activities resumed in Phase III, the original seasonal patterns were not fully restored.

Regarding ADV, we found that its positive rate was significantly suppressed in Phase I, and ADV recovered to near pre-epidemic levels in Phase II. In Phase III, ADV exhibited a significant recovery, with its total positive rate increasing by 180.65% compared with the pre-epidemic average. An epidemiological study in Beijing, China, demonstrated that strict NPIs led to a significant reduction in the positive rate of ADV in children, with the dominant types changing from ADV-B to ADV-C (46). The resurgence of ADV-associated ARTIs in northern Vietnam in the second half of 2022 further underscores the severity and persistence of this pathogen’s impact (47). These trends are similar to the epidemiological patterns of ADV observed in this study.

Interestingly, during Phase I, the annual positive rates of FluB, HPIV, HRV, and BoV were not significantly inhibited. However, the time series results of this study indicated that FluB, HPIV, HRV, and BoV were significantly suppressed during the most rigorous initial implementation. In fact, Suzhou (China) lowered its public health emergency level to level three at the end of March 2020. At this time, a few commercial activities were resumed and a few schools were reopened. We observed a gradual recovery of HRV in May 2020. On September 1, 2020, after the schools opened fully, we observed a recovery trend of FluB, HPIV, and BoV. Therefore, the positive rate of FluB, HRV, HPIV, and BoV during Phase I was higher than the average pre-pandemic level, possibly attributable to the rapid recovery of these viruses once the containment measures were slightly relaxed. With the adjustment of the zero-COVID policy, the positive rates of FluB and BoV were highest in Phase II, while HRV and HPIV showed an increasing trend in positive rates in Phases II and III. Additionally, although FluB briefly prevailed in Phase II, its positive rates remained low across all stages, indicating a limited threat to public health. A study from Italy noted the disappearance of a positive rate of FluB in children under 2 years old during the COVID-19 pandemic (48). Although wearing a mask can effectively block the transmission of influenza and coronavirus, its effectiveness against HRV is relatively poor (49). Zhang et al. found a sharp increase in hospital HRV infections in Beijing during the COVID-19 pandemic, especially among children aged 3 years and above, speculated to be related to the reopening of schools (50). Similarly, a study from Japan including 2,244 samples of patients with respiratory diseases reported an increase in the frequency of rhinovirus in children under 10 years old during the COVID-19 period (51). Liu et al. observed a significant increase in HRV and HPIV infections in 2020, especially after the reopening of schools, where the number of infections sharply increased. Compared with before the epidemic, the frequency of mixed infections decreased, consistent with the results of Phase I of this study (11). Additionally, our study found that the coinfection rate increased in 2023, with the coinfection rate of HRV and MP reaching 7.61%. In the first autumn after the relaxation of public restrictions, Finland experienced a sharp increase in HPIV cases, with the monthly positive rate in children aged 0–4 years six times higher than those aged more than 10 years (52). These results suggest that although the implementation of public health measures did not consistently significantly inhibit the positive rates of FluB, HRV, HPIV, and BoV, these pathogens may exhibit rebound characteristics after the relaxation of restrictions, potentially exceeding the average levels before the epidemic.

This study revealed a significant change in the positive rate of MP due to the COVID-19 pandemic. The positive rate of MP was significantly reduced during the entire COVID-19 pandemic period, and the resurgence of MP in 2023 was higher than the pre-pandemic level, suggesting that, unlike the recovery of other respiratory pathogens, the untypical delayed outbreak of MP occurred after the easing of the zero-COVID policy. Suzhou, China, experienced two peaks of MP infection in the summer and autumn of 2016 and 2019, respectively, indicating a cyclical epidemic pattern recurring once every 4 years. However, in 2020, following the implementation of COVID-19-related public health responses, the number of MP-positive cases notably decreased in the second and third quarters. Zhang et al.’s research attributed this decline to the restrictive measures and robust quarantine policies enforced by the Chinese government, resulting in a significant reduction in MP in 2020, particularly among children aged 3–6 years (53). Similarly, Ma et al.’s study indicated a statistically significant decrease in MP-positivity rate among children aged 1–6 years in 2020 and 2021 when compared with the pre-epidemic levels, suggesting a disruption in the original seasonal pattern (54). In our study, we found that, with the conclusion of China’s 3-year zero-COVID policy, the MP infection outbreak reached an unprecedented peak in 2023, with an annual positive rate soaring to 37.32%, which was particularly notable in children aged 8–16 years, with a staggering positive rate of 59.06%. This marked an annual growth rate of 116.72% compared with the pre-pandemic average, with the highest positive rate occurring in autumn. In contrast, the proportion of MP diagnoses and positive tests in the United States decreased during the pandemic, and while it rebounded in 2023, it has not reached pre-pandemic levels (55). This discrepancy suggests that the recovery of MP is influenced by various factors such as epidemic prevention and control strategies, social behavior patterns, and pathogen epidemic characteristics across different countries. The non-normal seasonal large-scale outbreak of MP infection in 2023 of this study may be attributed to the combined effect of the quadrennial epidemic cycle of MP and the “immune debt” accumulated after prolonged epidemic control measures. Therefore, after the easing of the zero-COVID policy, social activities gradually returned to normal, and it is still necessary to remain vigilant against MP infection, especially in cases of community-acquired pneumonia in autumn and winter.

Our study has several advantages. First, we analyzed the epidemic trend and positive rate of eight common respiratory pathogens covering the three stages of the COVID-19 pandemic in China from 2016 to 2023. To our knowledge, this has not been evaluated in previous studies in China, making our research the first study on this issue. Second, our study used a larger sample size of 82,708 children, providing us with sufficient statistical power to draw solid conclusions. Third, we used age and seasonal stratification to analyze the epidemic characteristics of respiratory pathogens in the three phases of the COVID-19 pandemic. Fourth, we applied advanced statistical methods, namely using time series analysis to predict the positive rate trends of various pathogens.

Nonetheless, our study has several limitations. First, the sample mainly consists of hospitalized children from a single center, the apparent changes in the positive rate of different pathogens may not reflect the true epidemiological trends in the prevalence of these pathogens. These changes may not be due solely attributable to the changes in COVID-19 control policy, but may also be related to the changes in the health-seeking behavior of communities. Our analysis is based on the positive testing rate, which could not be investigated for the modified health-seeking behavior of communities by using the current dataset alone. Second, the research is confined to Suzhou, China, which may restrict its geographical applicability. Third, the study only focused on detecting eight common respiratory pathogens, which could underestimate the true burden of infection.

## 5 Conclusion

We assessed the epidemic of respiratory pathogens in 82,708 children in China across different stages of the COVID-19 pandemic and described changes in their epidemiological characteristics. In the pandemic’s early stages, the strictest public health measures significantly reduced the positive rates of pathogens. The seasonal patterns and the age of onset of most pathogens changed due to the COVID-19 pandemic. With the gradual relaxation of measures, particularly after the 2023 easing of the zero-COVID policy, the positive rates of multiple pathogens not only recovered but also significantly surpassed the pre-epidemic levels. However, the seasonal epidemic trend of most pathogens has not fully returned to the pre-pandemic state. This recovery is attributed to increased social interaction, relaxed epidemic control measures, and changes in collective immunity, reflecting the “immune debt” effect from a long-term reduction of pathogen exposure. This phenomenon underscores the need to strengthen monitoring and control of respiratory pathogens in the post-pandemic period to mitigate health risks associated with immunization debts. Therefore, we advocate for careful consideration and reduction of the potential impact of immunization debt when formulating future public health strategies, alongside the adoption of comprehensive preventive measures.

## Data Availability

The raw data supporting the conclusions of this article will be made available by the authors, without undue reservation.
