# Supplementary material for: Inhibition, transition, and surge: dynamic evolution of pediatric respiratory pathogen trends amid COVID-19 pandemic policy adjustments
Source: Front Public Health. 2024 Aug 22;12:1420929. doi: 10.3389/fpubh.2024.1420929 (PMC11374627; doi:10.3389/fpubh.2024.1420929)
Supplement: Supplementary file 1 [file Data_Sheet_1.docx]

Supplementary Material

**
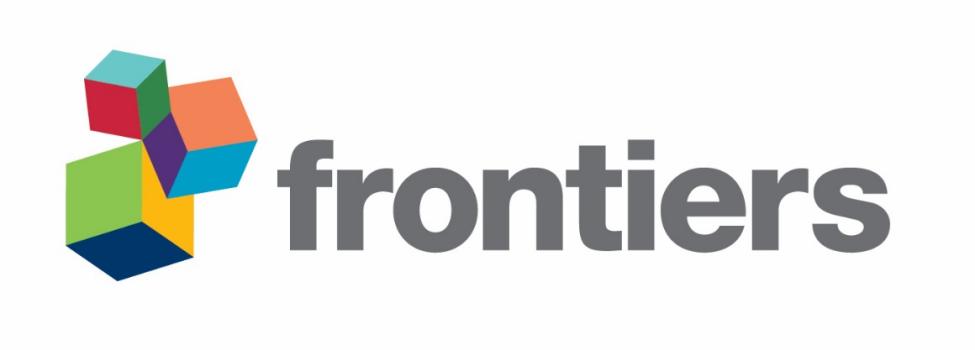
**

**Supplementary Figure 1. Monthly reported ARTI cases and research phase division in Suzhou 2016–2023.** On January 24, 2020, China implemented a national lockdown and level 1 public health emergency response, utilizing non-pharmaceutical interventions (NPIs). In 2020, Suzhou witnessed the strict enforcement of the zero-COVID policy. From 2021 to 2022, national NPIs were gradually eased while the dynamic zero-clearing policy was introduced. Finally, on December 27, 2022, China decided to end its zero-COVID policy. We have defined four periods from 2016 to 2023 according to the adjustments made to the zero-COVID policy in Suzhou: pre-pandemic (January 2016 to December 2019), phase I (January 2020 to December 2020), phase II (January 2021 to December 2022), and phase III (January to December 2023).


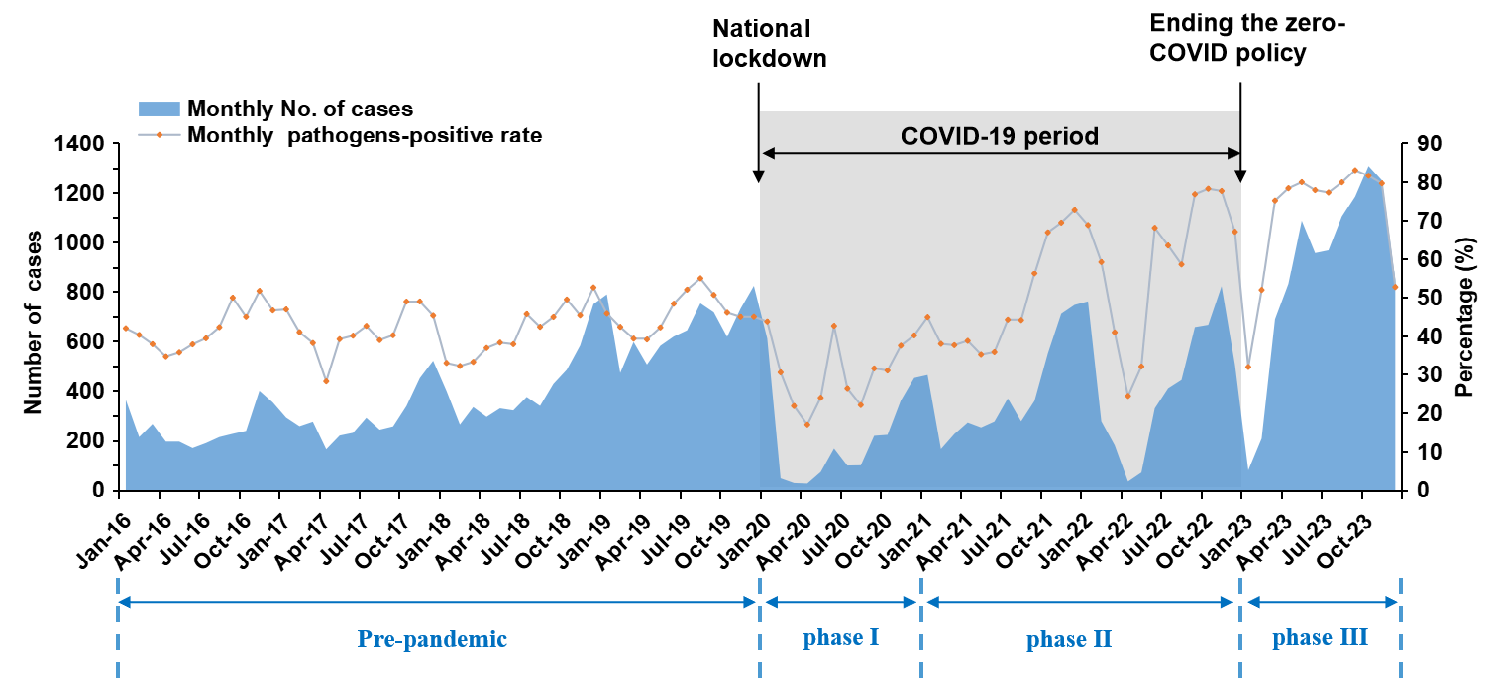


**Supplementary Figure 2. The proportion of pathogen infection in patients with ARTIs.** HRSV: human respiratory syncytial virus, FluA: influenza A, FluB: influenza B, HPIV: human parainfluenza virus, ADV: adenovirus, HRV: human rhinovirus, BoV: bocavirus, MP: mycoplasma pneumoniae.

**
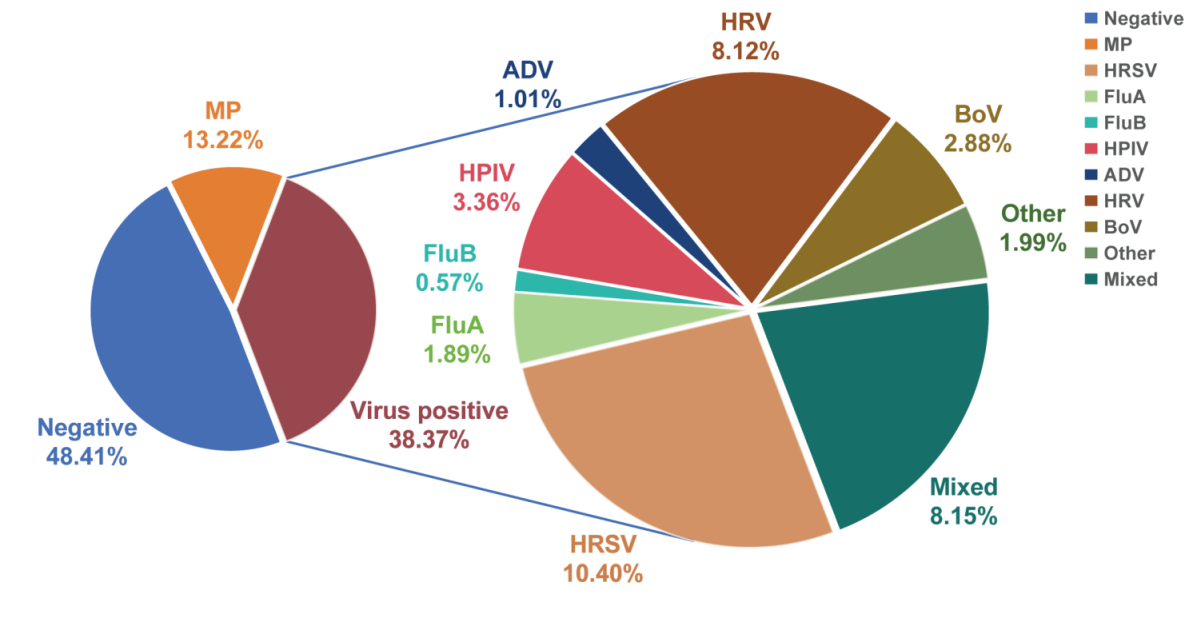
**

**Supplementary Table 1. Positive rate (%) of respiratory pathogens among pediatric inpatients among hospitalized children with ARTIs from 2016 to 2023.**

|  | **Pre-pandemic** | | | | **Phase I** | **Phase II** | | **Phase III** |
| --- | --- | --- | --- | --- | --- | --- | --- | --- |
|  | 2016 | 2017 | 2018 | 2019 | 2020 | 2021 | 2022 | 2023 |
| **HRSV** | 14.29 (1028/7193) | 14.50 (1223/8435) | 12.24 (1450/11846) | 8.22  (1411/17159) | 8.39  (580/6909) | 19.86 (1842/9273) | 7.36  (575/7814) | 16.01  (2254/14079) |
| **FluA** | 1.27  (91/7193) | 0.37  (31/8435) | 0.69  (82/11846) | 0.85  (145/17159) | 0.32  (22/6909) | 0.00  (0/9273) | 4.63  (362/7814) | 8.94  (1259/14079) |
| **FluB** | 0.21  (15/7193) | 0.53  (45/8435) | 0.46  (55/11846) | 0.33  (56/17159) | 0.71  (49/6909) | 1.24  (115/9273) | 2.38  (186/7814) | 0.83  (117/14079) |
| **HPIV** | 3.10  (223/7193) | 3.60  (304/8435) | 3.60  (427/11846) | 3.09  (530/17159) | 4.15  (287/6909) | 4.01  (372/9273) | 10.62  (830/7814) | 7.19  (1012/14079) |
| **ADV** | 1.27  (91/7193) | 1.65  (139/8435) | 1.04  (123/11846) | 1.86  (320/17159) | 0.41  (28/6909) | 0.74  (69/9273) | 2.10 (164/7814) | 3.49 (492/14079) |
| **HRV** | 10.30  (741/7193) | 10.35  (873/8435) | 9.02  (1069/11846) | 6.78  (1164/17159) | 11.41 (788/6909) | 15.42 (1430/9273) | 19.91  (1556/7814) | 21.24  (2991/14079) |
| **BoV** | 3.34  (240/7193) | 5.86  (494/8435) | 5.35  (634/11846) | 4.76  (817/17159) | 5.73  (396/6909) | 7.03  (652/9273) | 6.96  (544/7814) | 2.15  (302/14079) |
| **MP** | 13.93 (1002/7193) | 11.27  (951/8435) | 13.78 (1632/11846) | 23.90 (4101/17159) | 5.67  (392/6909) | 7.60  (705/9273) | 9.30 (727/7814) | 37.32  (5254/14079) |
| **Positive cases** | 41.76  (3044/7193) | 42.21  (3561/8435) | 41.58  (4926/11846) | 45.89  (7875/17159) | 35.10  (2425/6909) | 50.60  (4692/9273) | 66.29  (5180/7814) | 77.90  (10968/14079) |

The numerators in brackets are the total number of positive samples and the denominators are the total number of samples tested. HRSV: human respiratory syncytial virus, FluA: influenza A, FluB: influenza B, HPIV: human parainfluenza virus, ADV: adenovirus, HRV: human rhinovirus, BoV: bocavirus, MP: mycoplasma pneumoniae. Positive cases include both single and mixed respiratory infections.

**Supplementary Figure 3. Monthly positive cases and positive rates of respiratory pathogens among hospitalized children with ARTIs from 2016-2023.** (A) Positive cases and positive rates of HRSV. (B) Positive cases and positive rates of FluA. (C) Positive cases and positive rates of FluB. (D) Positive cases and positive rates of HPIV. (E) Positive cases and positive rates of ADV. (F) Positive cases and positive rates of HRV. (G) Positive cases and positive rates of BoV. (H) Positive cases and positive rates of MP.


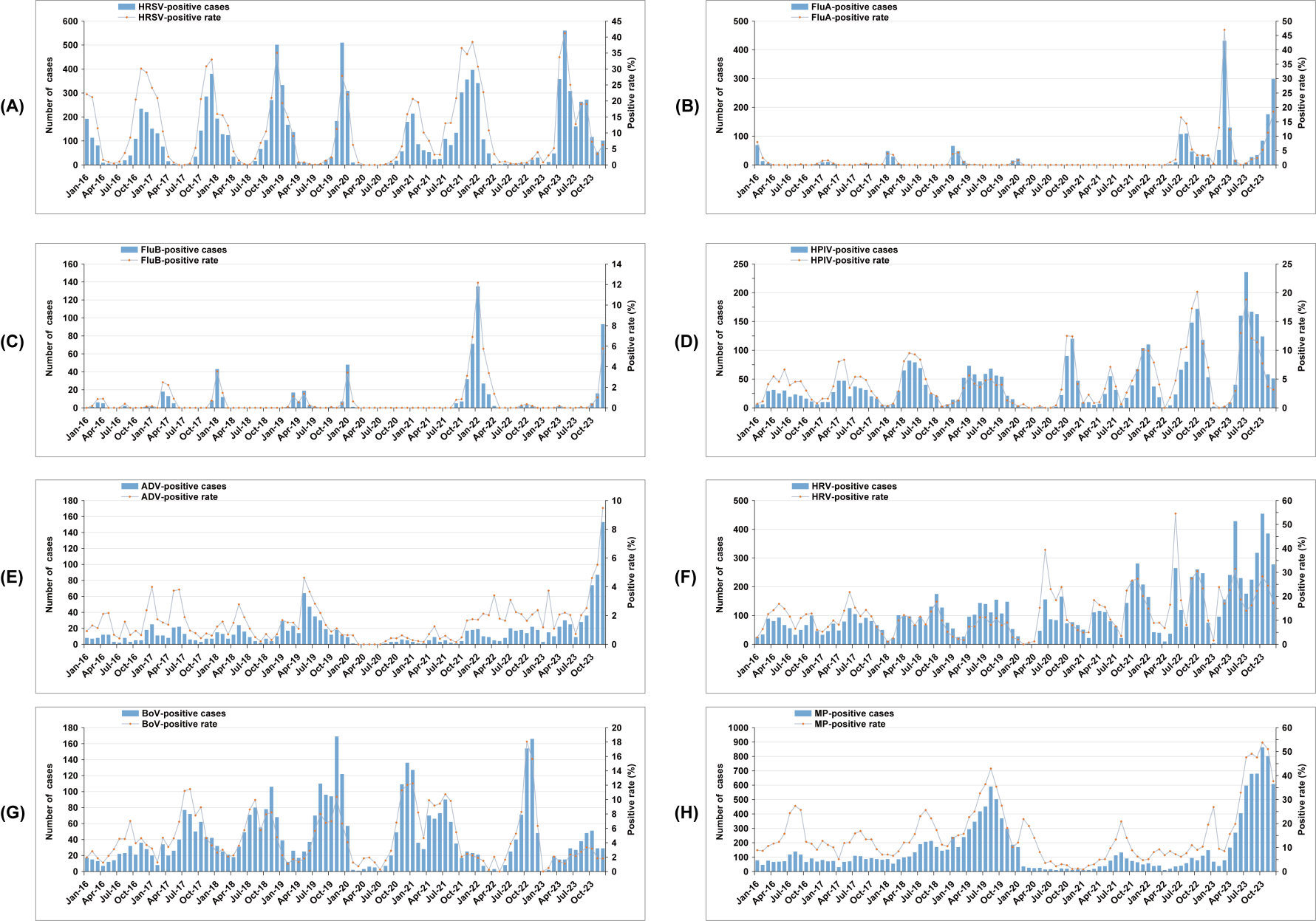


**Supplementary Table 2 Comparison of the coinfection patterns across pre-pandemic, Phase I, Phase II, and Phase III periods. Only the top 6 pairs of the coinfection viruses are listed.**

|  | **Pre-pandemic** | **Phase I** | | **Phase II** | | **Phase III** | |
| --- | --- | --- | --- | --- | --- | --- | --- |
| **Pathogens** | Coinfection rate (%) | Pathogens | Coinfection rate (%) | Pathogen | Coinfection rate (%) | Pathogens | Coinfection rate (%) |
| **HRV-MP** | 1.55 | HRV-BoV | 0.61 | HPIV-HRV | 1.40 | HRV-MP | 7.61 |
| **BoV-MP** | 0.95 | HRSV-BoV | 0.42 | HRSV-HRV | 1.31 | HRSV-HRV | 2.39 |
| **HRV-BoV** | 0.68 | HPIV-BoV | 0.39 | HRV-BoV | 1.24 | HPIV-MP | 2.25 |
| **HRSV-MP** | 0.63 | HPIV-HRV | 0.33 | HRV-MP | 1.10 | HRSV-MP | 1.59 |
| **HRSV-HRV** | 0.40 | HRSV-MP | 0.26 | HRV-HMPV | 0.71 | FluA-MP | 1.33 |
| **HPIV-MP** | 0.39 | HRV-MP | 0.23 | HRSV-BoV | 0.51 | HPIV-HRV | 1.29 |

**Supplementary Table 3 Comparison of the age distribution in respiratory pathogen infections across the pre-pandemic, Phase I, Phase II, and Phase III periods.**

|  | **Age Group** | **Total number**  **of cases(n)** | **HRSV** | **FluA** | **FluB** | **HPIV** | **ADV** | **HRV** | **BoV** | **MP** | **Positive case** |
| --- | --- | --- | --- | --- | --- | --- | --- | --- | --- | --- | --- |
|  |  |  | **Percentage of virus-positive specimens [n(%)]** | | | | | | | | |
| **Pre-pandemic** | 0～1 y | 22887 | 4012(17.53) | 132(0.58) | 38(0.17) | 1000(4.37) | 141(0.62) | 2020(8.83) | 1066(4.66) | 1700(7.43) | 9065(39.61) |
|  | 2～3 y | 10219 | 899(8.80) | 128(1.25) | 40(0.39) | 352(3.44) | 226(2.21) | 934(9.14) | 842(8.24) | 1759(17.12) | 4652(45.52) |
|  | 4～7 y | 9016 | 177(1.96) | 79(0.88) | 77(0.85) | 115(1.28) | 267(2.96) | 723(8.02) | 252(2.80) | 3043(33.75) | 4311(47.81) |
|  | 8～16 y | 2511 | 24(0.96) | 10(0.40) | 16(0.64) | 17(0.68) | 39(1.55) | 170(6.77) | 25(1.00) | 1193(47.51) | 1378(54.88) |
|  | χ^2^ | | 1971.810 | 47.393 | 84.941 | 250.698 | 284.936 | 19.781 | 415.692 | 4884.236 | 358.712 |
|  | *P* | | 0.000 | ＜0.001 | ＜0.001 | ＜0.001 | ＜0.001 | ＜0.001 | ＜0.001 | 0.000 | ＜0.001 |
| **Phase I** | 0～1 y | 3385 | 403(11.91) | 7(0.21) | 9(0.27) | 165(4.87) | 10(0.30) | 316(9.34) | 171(5.05) | 88(2.60) | 1096(32.38) |
|  | 2～3 y | 1779 | 142(7.98) | 4(0.22) | 18(1.01) | 87(4.89) | 7(0.39) | 254(14.28) | 165(9.27) | 67(3.77) | 714(40.13) |
|  | 4～7 y | 1297 | 31(2.39) | 6(0.46) | 14(1.08) | 34(2.62) | 11(0.85) | 167(12) | 58(4.47) | 143(11.03) | 462(35.62) |
|  | 8～16 y | 448 | 4(0.89) | 5(1.12) | 8(1.79) | 1(0.22) | 0(0) | 51(11.38) | 2(0.45) | 84(18.75) | 153(34.15) |
|  | χ^2^ | | 148.243 | 11.649 | 21.658 | 29.35 | 9.143 | 31.656 | 71.203 | 291.129 | 31.138 |
|  | *P* | | ＜0.001 | 0.009 | ＜0.001 | ＜0.001 | 0.027 | ＜0.001 | ＜0.001 | ＜0.001 | ＜0.001 |
| **Phase II** | 0～1 y | 6631 | 1327(20.01) | 57(0.86) | 49(0.74) | 437(6.59) | 50(0.75) | 969(14.61) | 333(5.02) | 132(1.99) | 3234(49.77) |
|  | 2～3 y | 4865 | 795(16.34) | 89(1.83) | 76(1.56) | 481(9.89) | 97(1.99) | 919(18.89) | 664(13.65) | 272(5.59) | 3213(66.04) |
|  | 4～7 y | 4231 | 252(5.96) | 177(4.18) | 124(2.93) | 247(5.84) | 70(1.65) | 898(21.22) | 190(4.49) | 608(14.37) | 2658(62.82) |
|  | 8～16 y | 1360 | 43(3.16) | 39(2.87) | 52(3.82) | 37(2.72) | 16(1.18) | 200(14.71) | 9(0.66) | 420(30.88) | 767(56.40) |
|  | χ^2^ | | 575.992 | 143.316 | 108.022 | 110.494 | 35.701 | 92.885 | 495.073 | 1496.434 | 401.934 |
|  | *P* | | ＜0.001 | ＜0.001 | ＜0.001 | ＜0.001 | ＜0.001 | ＜0.001 | ＜0.001 | 0.000 | ＜0.001 |
| **Phase III** | 0～1 y | 3105 | 1022(32.91) | 211(6.80) | 15(0.48) | 386(12.43) | 54(1.74) | 565(18.20) | 116(3.74) | 381(12.27) | 2269(73.07) |
|  | 2～3 y | 2581 | 650(25.18) | 241(9.34) | 14(0.54) | 261(10.11) | 118(4.57) | 710(27.51) | 134(5.19) | 615(23.83) | 2142(82.99) |
|  | 4～7 y | 5108 | 517(10.12) | 575(11.26) | 57(1.12) | 245(4.80) | 236(4.62) | 1166(22.83) | 49(0.96) | 2318(45.38) | 4075(79.77) |
|  | 8～16 y | 3285 | 65(1.98) | 232(7.06) | 31(0.94) | 120(3.65) | 84(2.56) | 550(16.74) | 3(0.09) | 1940(59.06) | 2482(73.91) |
|  | χ^2^ | | 1434.120 | 65.932 | 12.705 | 266.381 | 65.006 | 125.210 | 251.803 | 1839.124 | 101.784 |
|  | *P* | | 0.000 | ＜0.001 | 0.005 | ＜0.001 | ＜0.001 | ＜0.001 | ＜0.001 | 0.000 | ＜0.001 |

**Supplementary Table 4 Comparison of Seasonal distribution in respiratory pathogen infections across the pre-pandemic, Phase I, Phase II, and Phase III periods.**

| **Season** | **Period** | **Total number**  **of cases(n)** | **HRSV** | **FluA** | **FluB** | **HPIV** | **ADV** | **HRV** | **BoV** | **MP** |
| --- | --- | --- | --- | --- | --- | --- | --- | --- | --- | --- |
|  |  |  | **Percentage of pathogens-positive cases [n(%)]** | | | | | | |  |
| **Winter**  **(12m~2m)** | **Pre-pandemic** | 12711 | 3020(23.76) | 313(2.46) | 74(0.58) | 102(0.80) | 172(1.35) | 476(3.74) | 428(3.37) | 1310(10.31) |
|  | **Phase I** | 2689 | 498(18.52) | 22(0.82) | 49(1.82) | 52(1.93) | 15(0.56) | 95(3.53) | 195(7.25） | 218(8.11) |
|  | **Phase II** | 4842 | 1174(24.25) | 25(0.52) | 233(4.81) | 323(6.67) | 69(1.43) | 563(11.63) | 262(5.41) | 312(6.44) |
|  | **Phase III** | 2269 | 115(5.07) | 352(15.51) | 93(4.10) | 53(2.34) | 171(7.54) | 378(16.66) | 31(1.37) | 714(31.47) |
| **χ^2^** | | | 439.09 | 1309.364 | 378.144 | 527.645 | 439.687 | 767.038 | 150.621 | 1070.749 |
| ***P*** | | | ＜0.001 | ＜0.001 | ＜0.001 | ＜0.001 | ＜0.001 | ＜0.001 | ＜0.001 | ＜0.001 |
| **Spring**  **(3m~5m)** | **Pre-pandemic** | 10570 | 519(4.91) | 29(0.27) | 90(0.85) | 565(5.35) | 216(2.04) | 984(9.31) | 250(2.37) | 1544(14.61) |
|  | **Phase I** | 609 | 1(0.16) | 0(0.00) | 0(0.00) | 1(0.16) | 0(0.00) | 50(8.21) | 10(1.64) | 74(12.15) |
|  | **Phase II** | 2843 | 192(6.75) | 2(0.07) | 17(0.60) | 59(2.08) | 33(1.16) | 426(14.98) | 168(5.91) | 160(5.63) |
|  | **Phase III** | 3340 | 966(28.92) | 579(17.34) | 2(0.06) | 51(1.53) | 62(1.86) | 825(24.70) | 49(1.47) | 514(15.39) |
| **χ^2^** | | | 1782.957 | 2331.008 | 29.631 | 157.28 | 21.436 | 547.691 | 135.431 | 175.087 |
| ***P*** | | | ＜0.001 | 0.000 | ＜0.001 | ＜0.001 | ＜0.001 | ＜0.001 | ＜0.001 | ＜0.001 |
| **Summer**  **(6m~8m)** | **Pre-pandemic** | 9711 | 56(0.58) | 1(0.01) | 6(0.06) | 524(5.40) | 195(2.01) | 1086(11.18) | 665(6.85) | 2602(26.79) |
|  | **Phase I** | 1230 | 1(0.08） | 0(0.00) | 0(0.00) | 1(0.08) | 1(0.08) | 327(26.59) | 13(1.06) | 40(3.25) |
|  | **Phase II** | 4137 | 227(5.49） | 225(5.44) | 0(0.00) | 260(6.28) | 55(1.33) | 610(14.74) | 298(7.20) | 452(10.93) |
|  | **Phase III** | 3866 | 731(18.91) | 34(0.88) | 1(0.03) | 563(14.56) | 62(1.60) | 631(16.32) | 94(2.43) | 1681(43.48) |
| **χ^2^** | | | 1905.358 | 662.503 | 3.734 | 464.265 | 28.954 | 244.88 | 168.718 | 1459.861 |
| ***P*** | | | 0.000 | ＜0.001 | 0.292 | ＜0.001 | ＜0.001 | ＜0.001 | ＜0.001 | 0.000 |
| **Autumn**  **(9m~11m)** | **Pre-pandemic** | 11641 | 1517(13.03) | 6(0.05) | 1(0.01) | 293(2.52) | 90(0.77) | 1301(11.18) | 842(7.23) | 2230(19.16) |
|  | **Phase I** | 2381 | 80(3.36) | 0(0.00) | 0(0.00) | 232(9.74) | 12(0.50) | 316(13.27) | 178(7.48) | 50(2.10) |
|  | **Phase II** | 5265 | 824(15.65) | 110(2.09) | 51(0.97) | 561(10.66) | 76(1.44) | 1387(26.34) | 468(8.89) | 508(9.65) |
|  | **Phase III** | 4604 | 442(9.60) | 294(6.39) | 21(0.46) | 345(7.49) | 197(4.28) | 1157(25.13) | 128(2.78) | 2345(50.93) |
| **χ^2^** | | | 271.91 | 832.363 | 120.423 | 530.929 | 284.575 | 825.823 | 159.531 | 3330.149 |
| ***P*** | | | ＜0.001 | ＜0.001 | ＜0.001 | ＜0.001 | ＜0.001 | ＜0.001 | ＜0.001 | 0.000 |
